# Biological Activities of Two Major Copaiba Diterpenoids and Their Semi-synthetic Derivatives

**DOI:** 10.1007/s43450-020-00002-y

**Published:** 2020-02-21

**Authors:** Serhat Sezai Çiçek, Arlette Wenzel-Storjohann, Ulrich Girreser, Deniz Tasdemir

**Affiliations:** 1 Pharmazeutisches Institut, Abteilung Pharmazeutische Biologie, Christian-Albrechts-Universität zu Kiel, Kiel, Germany; 2 GEOMAR Centre for Marine Biotechnology, Research Unit Marine Natural Products Chemistry, GEOMAR Helmholtz Centre for Ocean Research Kiel, Kiel, Germany; 3 Pharmazeutisches Institut, Abteilung Pharmazeutische und Medizinische Chemie, Christian-Albrechts-Universität zu Kiel, Kiel, Germany; 4 Christian-Albrechts-Universität zu Kiel, Kiel, Germany

**Keywords:** Natural product, Partial synthesis, Traditional medicine, Diterpene acid, MRSA, NMR

## Abstract

The oleoresin of *Copaifera reticulata* Ducke, Fabaceae, is a traditional Brazilian remedy used for a wide range of applications. Commonly named copaiba, the oleoresin has been found to exhibit strong antimicrobial effects in our previous study, which could be attributed to some of its diterpenoid constituents. In order to find new biological activities and to eventually enhance the before observed effects, (−)-polyalthic acid (**1**) and kaurenoic acid (**2**), together with eight prepared semi-synthetic derivatives (**1a**–**1c** and **2a**–**2e**) were evaluated for their cytotoxic, antibacterial and antifungal properties. Regarding the gram-positive bacteria *Enterococcus faecium* and methicillin-resistant *Staphylococcus aureus*, we found that both the exocylic methylene group and the carboxyl group were crucial for the activity against these two clinically relevant bacterial strains. Investigation of the antifungal activity, in contrast, showed that the carboxyl group is unnecessary for the effect against the dermatophytes *Trichophyton rubrum* and *Cryptococcus neoformans*, indicated by low micromolar IC_50_ values for both (−)-polyalthic acid diethylamide (**1a**) as well as (−)-polyalthic acid methyl ester (**1b**). Apart from studying the biological activity, the structure of one semi-synthetic derivative, compound **1c**, is being reported for the first time. During the course of the structure elucidation of the new compound, we discovered inconsistencies regarding the stereochemistry of polyalthic acid and its stereoisomers, which we clarified in the present work.

**Electronic supplementary material:**

The online version of this article (10.1007/s43450-020-00002-y) contains supplementary material, which is available to authorized users.

**Graphical Abstract:**
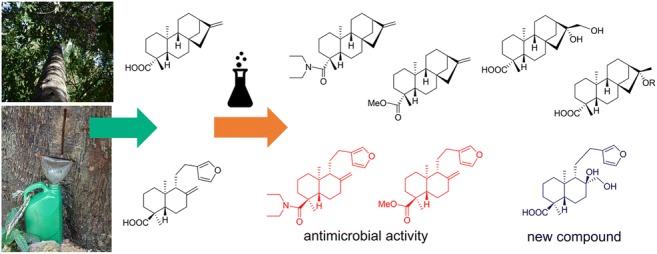
.

## Introduction

Copaiba, or copaiba oil, is the name of an oleoresin obtained from selected species of the genus *Copaifera*, Fabaceae (Leandro et al. 2012). Copaiba is widely used in the traditional medicine in Brazil and other Latin American countries for treatment of various diseases, such as skin and urinary tract infections, respiratory diseases, ulcers, rheumatism, herpes, tumours and tetanus disease (Ohsaki et al. 1994). Due to its antibacterial, antihelminthic, trypanocidal and leishmanicidal applications, the oleoresin represents an important natural remedy for people without access to modern medicine and commercial drugs (Ohsaki et al. 1994; Leandro et al. 2012).

Copaiba consists of sesquiterpenes and diterpenes, the latter usually being present as diterpene acids (Leandro et al. 2012). Sesquiterpenes represent the main compounds that can count up to 98% of the oleoresin by weight, and show a rather stable metabolite pattern, with β-caryophyllene being the major constituent in most *Copaifera* species (Leandro et al. 2012). However, β-bisabolene has been identified as the major sesquiterpene of *Copaifera reticulata* Ducke collected in the Pará region (northern Brazil) (Pfeifer Barbosa et al. 2019). In contrast to the sesquiterpenes, copaiba diterpenoids show much higher interspecific variation and therefore attracted particular interest in recent years due to varying biological activities observed for the different *Copaifera* species (Leandro et al. 2012). These activities comprise cytotoxic, antibacterial, antifungal and antiprotozoal properties as part of numerous *in vitro* and *in vivo* studies attempting to attribute the detected effect to certain compounds or compound classes.

The cytotoxic potential of the copaiba oleoresin seems to mainly derive from its sesquiterpene constituents, as respective investigations on diterpenoids resulted in weak to moderate (but then non-selective) cytotoxic effects (Cavalcanti et al. 2009; Pfeifer Barbosa et al. 2019). The only exception is kolavenol, which showed antitumour effects against IMC carcinoma in mice, determined by an increase of life span of the animals (Ohsaki et al. 1994). In contrast, the main sesquiterpenes showed selective moderate (β-bisabolol) to pronounced (β-caryophyllene and β-caryophyllene oxide) cytotoxic activities (Kubo et al. 1996; Yeo et al. 2016). With respect to their higher concentration in the oleoresin, it is more likely that sesquiterpenes are responsible for the antitumour properties of copaiba (Pfeifer Barbosa et al. 2019).

On the other hand, copaiba diterpenoids showed good in vitro activities against causative agents of infectious parasitic diseases. Copalic acid, 3β-hydroxycopalic acid and methyl copalate, for example, inhibited the growth of the amastigote forms of *Trypanosoma cruzi*, whereas kaurenoic acid (**2**) and some of its semi-synthetic derivatives were found active against its erythrocytic trypomastigote forms (Vieira et al. 2002; Izumi et al. 2012). (−)-Polyalthic acid (**1**), another major copaiba diterpenoid showed activity against *T. brucei* and amastigote forms of *Leishmania donovani* (Mizuno et al. 2015). Also here, semi-synthetic derivatives were prepared, of which some showed comparable to slightly higher activities than the natural product. However, neither (−)-polyalthic acid (**1**) nor any of its derivatives was leishmanicidal against promastigote forms of *L. donovani*.

Moreover, copaiba diterpenoids revealed antibacterial activity against both gram-positive and gram-negative bacteria, with pronounced effects against *Bacillus subtilis* and five different *Streptococcus* species (Tincusi et al. 2002; Souza et al. 2011). In our previous study, we reported strong inhibitory effects against the two clinically relevant bacterial strains methicillin-resistant *Staphylococcus aureus* (MRSA) and *Enterococcus faecium* for three diterpene acids, of which one was kaurenoic acid (**2**) (Pfeifer Barbosa et al. 2019). In the same study, antidermatophytic activity against two *Trichopyhton* species was detected for (−)-polyalthic acid (**1**), the major diterpenoid in the *Copaifera reticulata* oleoresin, along with weak [(−)-polyalthic acid, **1**] to moderate (kaurenoic acid, **2**) cytotoxic effects against six cancer cell lines. These findings prompted us to prepare semi-synthetic derivatives of both compounds in an attempt to enhance their antimicrobial and cytotoxic properties and to get more insights into their structure-activity-relationships (SAR). Furthermore, additional screenings were performed to discover new lead compounds against both human and plant pathogens.

## Materials and Methods

### Plant Material and Chemicals

(−)-Polyalthic acid (**1**) and kaurenoic acid (**2**) were isolated from the oleoresin of *Copaifera reticulata* Ducke, Fabaceae, as described in our previous studies (Çiçek et al. 2018; Pfeifer Barbosa et al. 2019). *N*-Chlorosuccinimide (98%) and triphenylphosphine (ReagentPlus, 99%), sulphuric acid (puriss., analytical grade) and LC-MS grade formic acid were purchased from Sigma-Aldrich Co., St. Louis, MO, USA. Hydrochloric acid (25%, analytical grade) was obtained from Honeywell, Seelze, Germany, while sodium hydroxide solution (2N) was purchased from Carl Roth GmbH, Karlsruhe, Germany. Diethylamine (for synthesis), acetone (analytical grade), methanol (gradient grade or LC-MS grade) and water (LC-MS grade) as well as other analytical grade solvents used for purification were obtained from VWR International GmbH, Darmstadt, Germany. Solid phase extraction (SPE) columns (Chromabond SB 3 ml/500 mg) were obtained from Macherey-Nagel GmbH & Co. KG, Düren, Germany. Deuterated methanol (Lot P3021, 99.80%), deuterated DMSO (Lot S1051, 99.80%) and deuterated chloroform (Lot Q1981, 99.80%) for NMR spectroscopy were obtained from Eurisotop GmbH, Saarbrücken, Germany. Conventional 5-mm sample tubes were purchased from Rototec-Spintec GmbH, Griesheim, Germany.

### General Experimental Procedures

Semi-synthetic derivatives of compounds **1** and **2** were prepared in 5-ml V-Vials (Wheaton, Millville, NJ, USA) using a RET basic magnetic stirrer with integrated heater (IKA-Werke GmbH & Co. KG, Staufen, Germany). LC-DAD-ELSD analysis was accomplished as stated in Çiçek et al. (2018); LC-MS and GC-MS analyses were conducted as described in Pfeifer Barbosa et al. (2019). High-resolution MS spectrum was recorded on micrOTOF II-High-performance TOF-MS system (Bruker®, Billerica, MA, USA) equipped with an electrospray ionisation source. Specific rotation of the compounds measured in methanol on a Jasco P-2000 polarimeter (Jasco, Pfungstadt, Germany). NMR spectra were recorded using a Bruker Avance III 400 NMR spectrometer operating at 400 MHz for the proton channel and 100 MHz for the ^13^C channel with a 5 mm PABBO broad band probe with a z gradient unit at 293 K (Bruker BioSpin GmbH, Rheinstetten, Germany). Reference values were 3.31 (^1^H) and 49.15 ppm (^13^C) for methanol as well as 2.50 (^1^H) and 39.51 ppm (^13^C) for DMSO, respectively.

**Table 1:** NMR data for compound **1c** in methanol-*d*_4_ and DMSO-*d*_6_ (400 MHz, *δ* in ppm, *J* in Hz)

Position	^1^H NMR (CD_3_OD)	^13^C NMR (CD_3_OD)	^1^H NMR (DMSO-*d*_6_)	^13^C NMR (DMSO-*d*_6_)
1	1.77 (m)	41.0	1.63 (m)	38.4
	1.14 (m)		0.97 (m)	
2	1.68 (m)	19.1	1.54 (m)	16.8
	1.59 (m)		1.46 (m)	
3	2.24 (m)	38.7	2.06 (m)	36.8
	1.29 (m)		1.06 (m)	
4		48.6		46.4
5	1.89 (s)	50.3	1.68 (s)	49.9
6	1.47 (m)	24.4	1.31 (m)	22.0
	1.27 (m)		1.05 (m)	
7	1.81 (m)	38.8	1.64 (m)	36.1
	1.58 (m)		1.45 (m)	
8		76.8		74.4
9	1.42 (s)	62.8	1.25 (s)	60.6
10		39.7		37.6
11	1.87 (m)	27.5	1.74 (m)	25.0
	1.46 (m)		1.30 (m)	
12	2.63 (m)	29.9	2.35 (m)	27.5
	2.48 (td, 12.1, 3.9)		2.06 (m)	
13		126.1		125.6
14	6.35 (d, 0.9)	112.5	6.36 (d, 0.8)	111.3
15	7.39 (t, 1.4)	144.2	7.53 (t, 1.5)	142.8
16	7.30 (s)	140.3	7.42 (s)	138.6
17	3.65 (d, 10.8)	64.1	3.38 (d, 10.7)	61.3
	3.51 (d, 7.9)		3.27 (d, 10.7)	
18		n.o.		180.6
19	1.16 (s)	17.5	1.01 (s)	16.0
20	0.85 (s)	17.3	0.72 (s)	15.5

*n.o.*, not observed

### Preparation of Derivatives

#### Carboxyl Amides

The compound (100 μmol) and triphenylphosphine were dissolved in 2 ml of dichloromethane, and the mixture was cooled in an ice bath. After adding 110 μmol of *N*-chlorosuccinimide in small portions, the mixture was vigorously stirred for 30 min at room temperature. Diethylamine (110 μmol) in 100 μmol of pyridine was added, and the mixture was stirred for another 10 min at room temperature. The mixture was subsequently concentrated under reduced pressure, and the by-products were removed by filtration and solid phase extraction using hexane as solvent to afford 6.2 mg of (−)-polyalthic acid-*N*,*N*-diethylamide (**1a**) and 7.5 mg kaurenoic acid-*N*,*N*-diethylamide (**2a**).

#### Carboxyl Esters

The formation of ester was accomplished in the same manner except that diethylamine was substituted with methanol in the last reaction step. This reaction yielded 4.2 mg (−)-polyalthic acid methyl ester (**1b**) and 7.3 mg kaurenoic acid methyl ester (**2b**).

#### Dihydroxy Derivatives

The compound (100 μmol) was dissolved under heating in a mixture of 800 μl of water and 200 μl of 2 M sodium hydroxide. The solution was subsequently cooled to 5 °C, and 250 mg of ice was added before adding a cold solution of 20.5 mg potassium permanganate in 500 μl of water over a period of 30 min. The mixture was kept at 0 to 5 °C for another 60 min before the precipitate was filtered off and washed with hot water. The filtrate was acidified with acetic acid, and the resulting precipitate was filtered of and washed with cold water and dried to afford 8.8 mg of (4*S*,8*S*)-15,16-epoxy-8,17-dihydroxy-13(16),14-*ent*-labdadien-18-oic acid [(1*S*,4*aR*,5*S*,6*S*,8*aS*)-5-[2-(furan-3-yl)ethyl]-1,4*a*-dimethyl-6-hydroxy-6-hydroxymethyl-3,4,5,7,8,8*a*-hexahydro-2*H*-naphthalene-1-carboxylic acid] (**1c**) and 13.0 mg of 16α,17-dihydroxy-*ent*-kauran-19-oic acid (**2c**), respectively, after recrystallisation from a mixture of hexane and *tert*-butyl methyl ether.

#### Monohydroxy Derivative

Compound **2** (50 mg) was dissolved in a mixture of 1500 μl acetone, 350 μl water and 150 μl hydrochloric acid (25%) and kept at 56 °C for 2 h under stirring. The solution was then transferred to a beaker containing 10 ml of water ,and the resulting precipitate was filtered and recrystallised from acetone to obtain 12.2 mg of 16α-hydroxy-*ent*-kauran-19-oic acid (**2d**).

#### Methoxy Derivative

Compound **2** (50 mg) was dissolved in a mixture of 2500 μl methanol and 150 μl sulphuric acid and kept at 65 °C for 3 h under stirring. The solution was subsequently cooled to − 18 °C and the resulting precipitate was filtered and recrystallised from methanol to obtain 7.9 mg of 16α-methoxy-*ent*-kauran-19-oic acid (**2e**).

### Biological Activity Testing

#### Antibacterial and Antiyeast Activity

The samples were tested against the ESKAPE panel of multidrug resistant bacterial human pathogens, including the gram-positive bacteria *Enterococcus faecium* (DSM 20477) and methillicin-resistant *Staphylococcus aureus* (MRSA, DSM 18827), and the gram-negative bacteria *Klebsiella pneumoniae* (DSM 30104), *Acinetobacter baumannii* (DSM 30007), *Pseudomonas aeruginosa* (DSM 1128) and *Escherichia coli* (DSM 1576). Furthermore the activity of the samples against four phytopathogenic bacteria, *Pseudomonas syringae* (DSM 50252), *Xanthomonas campestris* (DSM 2405), *Erwinia amylovora* (DSM 50901) and *Ralstonia solanacearum* (DSM 9544)*,* and against two human pathogen yeasts, *Candida albicans* (DSM 1386) and *Cryptococcus neoformans* (DSM 6973), was carried out. All test strains were purchased from Leibniz Institute DSMZ-German Collection of Microorganisms and Cell Cultures, Braunschweig, Germany.

The bacteria were cultivated in TSB medium (1.2% tryptic soy broth, 0.5% NaCl), except *E. faecium* which was cultivated in M92 medium (3% trypticase soy broth, 0.3% yeast extract, pH 7.0–7.2) and *R. solanacearum* which was grown in M186 (1% glucose, 0.5% peptone from soymeal, 0.3% malt extract, 0.3 yeast extract). The cultivation of *C. albicans* took place in M186/3 (0.3% glucose, 0.17% peptone from soymeal, 0.1% malt extract, 0.1% yeast extract) and for *C. neoformans* M186 was used as well. Overnight cultures of the test organisms were prepared and diluted to an optical density (600 nm) of 0.01–0.03. To prepare the assay, the test samples (20 mg/ml DMSO stock solution) were dissolved in medium and transferred into a 96-well microtiter plate and 200 μl of the cell suspension cultures was added to each well. The final assay concentration of the substances was 100 μg/ml. The inoculated microplates were incubated for 5 h at 37 °C and 200 rpm (*E. faecium* without shaking), or 28 °C and 200 rpm for 7 h for the phytopathogen bacteria and *C. neoformans*, respectively. To detect the inhibitory effect of the substances 10 μl of a resazurin solution (0.3 mg/ml phosphate-buffered saline) was added to the microplates and incubated again for 5–30 min before the fluorescence signal (560 nm/590 nm) was measured using the microplate reader (Tecan Infinite M200). For *E. faecium* the pH indicator bromocresol purple was used to determine the acidification caused by growing, and for *R. solanacearum* and *C. neoformans* the optical density at 600 nm after incubation time was recorded using the microplate reader as well. The resulting values were compared with a positive control (chloramphenicol for the bacteria, except *R. solanacearum* where tetracycline was used, nystatin for *C. albicans* and amphotericin B for *C. neoformans*) and a negative control (no compound) on the same plate. For IC_50_ determination, a dilution series was prepared and the IC_50_ value was calculated by Excel or GraphPad Prism as the concentration that shows 50% inhibition of viability on the basis of a negative control (no compound).

#### Cytotoxic Activity and Activity Against Dermatophytic Fungi

Measurements of cytotoxic activity and activity against dermatophytic fungi were performed as described in our previous study (Pfeifer Barbosa et al. 2019).

## Results and Discussion

### Preparation of Semi-synthetic Derivatives **1a**–**1c** and **2a**–**2e**

Semi-synthetic derivatives of (−)-polyalthic acid (**1**) and kaurenoic acid (**2**) were prepared on a small scale using 5-ml V-Vials and applying between 30 and 50 mg of diterpene acid. Derivatisations included the carboxyl group and the exocyclic methylene group, respectively. Thus, *N*,*N*-diethyl amide (**1a** and **2a**) and methyl ester (**1b** and **2b**) as well as dihydroxy derivatives (**1c** and **2c**) of both parent molecules were prepared as described in the Experimental section. Additionally, one monohydroxy derivative (**2d**) and one methoxy derivative (**2e**) of kaurenoic acid were prepared. All products were analysed by TLC using different mixtures of dichloromethane and ethanol as solvent and vanillin-sulphuric acid as spray reagent. Amides and esters were additionally checked by GC-MS^2^, whereas the other derivatives were analysed by UHPLC-DAD/ELSD and UHPLC-MS^2^. For structure elucidation, one- and two-dimensional NMR experiments were performed.
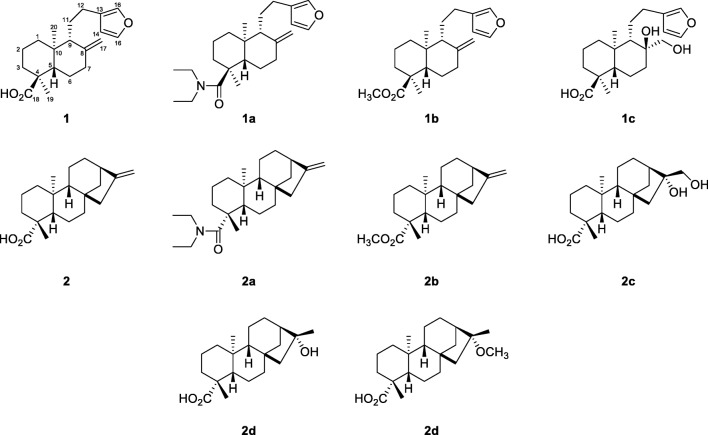


#### Amides and Methyl Esters

Amidation and esterification of diterpenoids was accomplished using *N*-chlorosuccinimide and triphenylphosphine for conversion of the carboxylic acids and adding either *N*,*N*-diethylamine or methanol in pyridine to the reaction mixture as described in the protocols of Frøyen (1994, 1995). The experiments were therefore downsized to 100 μmol of reactants (instead of 5 mmol) except for dichloromethane, of which 2 ml (instead of 6 ml) was used. Purification was conducted as described in the protocols (concentration, filtration and washing with diethyl ether, silica gel column with ether as eluent). However, for the last purification step another silica gel column with *n*-hexane as eluent was preferred over crystallisation or distillation, respectively. All four derivatives were subsequently analysed by GC-MS^2^ showing the expected *m/z* ratios of 371 (**1a**), 357 (**2a**), 330 (**1b**), and 316 (**2b**) and by NMR spectroscopy comparing their spectra to literature data (Narayanan and Venkatasubramanian 1965; Vieira et al. 2002; Mizuno et al. 2015; Santos et al. 2016). The methyl esters (**1b** and **2b**) were additionally identified by comparing their mass spectra to the spectra available in the NIST database.

#### Dihydroxy Derivatives

Dihydroxy derivatives of compounds **1** and **2** were prepared with potassium permanganate in alkaline medium following a protocol for the hydroxylation of lambertianic acid (Chernov et al. 2006). Lambertianic acid is an epimer of (+)-polyalthic acid at position C-4 and thus a stereoisomer of (−)-polyalthic acid (**1**), showing different configurations at positions C-5, C-9, and C-10. Using scales of 100 μmol of diterpenoid instead of 20 mmol, the reaction yielded (4*S*,8*S*)-15,16-epoxy-8,17-dihydroxy-13(16),14-*ent*-labdadien-18-oic acid (**1c**), a previously undescribed compound, and the known compound 16α,17-dihydroxy-*ent*-kauran-19-oic acid (**2c**). Whereas the latter compound was identified by comparison of MS and NMR spectra to literature data (Song et al. 2018), structure elucidation of compound **1c** is described in “Structure Elucidation and Configuration of Compound **1c**”.
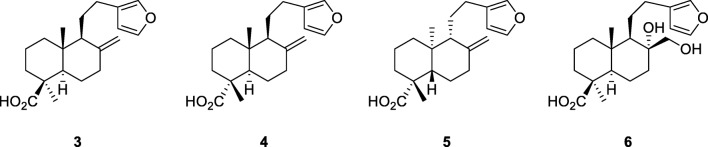


#### Monohydroxy and Methoxy Derivatives

Preparation of 16α-hydroxy-*ent*-kauran-19-oic acid (**2d**), 16α-methoxy-*ent*-kauran-19-oic acid (**2e**) was performed using acid-catalysed hydroxylation and methoxylation, respectively (Cavalcanti et al. 2009). For these reactions, amounts of 50 mg were applied instead of 1 g and the resulting products were identified by comparison of their MS and NMR data to literature (Chen et al. 2000; Yaouba et al. 2018).

### Structure Elucidation and Configuration of Compound **1c**

As mentioned above, compound **1c** was synthesised using a protocol (Chernov et al. 2006) reported for the transformation of lambertianic acid, a stereoisomer of (−)-polyalthic acid (**1**). The side chain at C-10 of lambertianic acid is β-oriented leading to α-oriented hydroxylation at position C-8, due to steric hindrance by the furanoethyl group. In contrast, (−)-polyalthic acid (**1**) shows an α-oriented side chain at C-10; hence, the resulting hydroxy group at position C-8 had to have β-orientation and the hydroxymethylene group α-orientation instead. The α-orientation of the hydroxymethylene group was confirmed using NOESY, where NOE correlations were observed for the protons at C-17 on both the methylene group at C-11 and the C-20 methyl group. Thus, the structure was identified as (4*S*,8*S*)-15,16-epoxy-8,17-dihydroxy-13(16),14-*ent*-labdadien-18-oic acid or (*rel*-1*S*,4*aR*,5*S*,6*S*,8*aS*)-5-[2-(furan-3-yl)ethyl]-1,4*a*-dimethyl-6-hydroxy-6-hydroxymethyl-3,4,5,7,8,8*a*-hexahydro-2*H*-naphthalene-1-carboxylic acid, respectively (see “Stereochemistry of Polyalthic Acid, Daniellic Acid and Lambertianic Acid”). Same as (−)-polyalthic acid (**1**), the new compound (**1c**) showed a negative specific rotation value [α_D_^20^–14.9, *c* = 0.1, methanol). HR-ESI-MS revealed *m/z* ratios of 373.1994 [*M* + Na]^+^ and 723.4090 [2 *M* + Na]^+^, corresponding to the molecular formula C_20_H_30_O_5_ with the experimental mass of *M* 350.2096 (calculated mass was *M* 350.2093). NMR data for compound **1c** is given in Table 1. 

### Stereochemistry of Polyalthic Acid, Daniellic Acid and Lambertianic Acid

As described above, hydroxylation of (−)-polyalthic acid (**1**) followed a protocol of Chernov et al. (2006), who used lambertianic acid as starting material. However, during the course of the structure determination of compound **1c** and assignment of the absolute configuration, some inconsistencies have been observed with regard to the stereochemistry of polyalthic acid and its structural isomers, which we attempted to resolve hereafter.

The first two of these four compounds to be reported were (−)-polyalthic acid (**1**) and daniellic acid in 1961. As the absolute configuration has not been determined yet, (−)-polyalthic acid is correctly described as (*rel-1S,4aS,5R,8aS*)-5-[2-(furan-3-yl)ethyl]-1,4*a*-dimethyl-6-methylene-decahydronaphthalene-1-carboxylic acid. Daniellic acid was isolated from *Daniellia oliveri* (Fabaceae, Caesalpinioideae) (Haeuser and Ourisson 1961), whereas (−)-polyalthic acid (**1**) was obtained from *Polyalthia fragrans* (Annonaceae) and named polyalthic acid (Gopinath et al. 1961). Both compounds were attributed to possess the “wrong” configuration corresponding to an *ent*-labdane scaffold and showing negative optical rotation values. Both compounds are epimers, only differing in the orientation of the carboxyl group at position C-4, which is axial for daniellic acid and equatorial for (−)-polyalthic acid (**1**). Five years later, the first report for lambertianic acid, isolated from *Pinus lambertiana* (Pinaceae), was made (Dauben and Dauben and German 1965). Lambertianic acid was found to be the optical antipode of daniellic acid and thus to be the first furan diterpene with the so-called normal labdane configuration. The last of the four isomers was the optical antipode of (−)-polyalthic acid (**1**), isolated from *Sequoia semperivirens*, Taxodiaceae (Ohta and Nawamaki 1978). However, instead of choosing an original trivial name (e.g. semperiviric acid), the authors named the compound (+)-polyalthic acid, being (*rel-1R,4aR,5S,8aR*)-5-[2-(furan-3-yl)ethyl]-1,4*a*-dimethyl-6-methylene-decahydronaphthalene-1-carboxylic acid. Thus, from then onwards, two forms of polyalthic acid existed in the literature. Except for the last 3 years, where (+)- and (−)- or *ent*- prefixes were used to differentiate between the two enantiomers (Bardají et al. 2016; Borges et al. 2016; Carneiro et al. 2018; Çiçek et al. 2018; Pfeifer Barbosa et al. 2019; Senedese et al. 2019), only one publication in almost four decades adopted such prefixes (Miyazawa et al. 1995). The missing stereochemistry did not represent a problem as long as the studies were dealing with *Copaifera*, because the *ent*-configuration is abundant in this genus (Leandro et al. 2012). However, some studies investigated plant species from other plant families; wherefore, the identity of the right enantiomer remained unknown. Thus, reporting the right configuration for polyalthic acid is absolutely necessary.

Another problem is that the introduction of (+)-polyalthic acid and the recent use of the *ent*-prefix for differentiation of the enantiomers seems to have caused confusions in some large databases, such as SciFinder™ or Reaxys™. For example, when searching for structure templates of polyalthic acid in the Reaxys database, three suggestions are delivered, of which two display (−)-polyalthic acid and the other one shows (+)-polyalthic acid. However, by using the name *ent*-polyalthic acid, also two structures are displayed, one for (−)-polyalthic acid and the second one for daniellic acid. For both compounds their semisystematic names are given along with twelve references per compound, thus giving equal priority to the correct and the incorrect structure. By looking for chemical structures in the SciFinder database, the name polyalthic acid leads to the structure of (−)-polyalthic acid, which is historically correct, whereas the search for *ent*-polyalthic acid gives the structure of daniellic acid and thus the wrong compound. Alas, these inconsistencies or confusions, respectively, led to several publications showing the structure of daniellic acid instead of (−)-polyalthic acid (Leandro et al. 2012, Çiçek et al. 2018, Da Silva et al. 2017; Pfeifer Barbosa et al. 2019). The mentioned problems can be partly dealt with by using the prefixes of the respective optical rotations, leading to the exact structures for both (+)- and (−)-polyalthic acid in the Reaxys database and also to the correct structure for (+)-polyalthic acid with the SciFinder database. Only the name (−)-polyalthic acid does not yield any results with SciFinder, but as the name polyalthic acid leads to the structure of (−)-polyalthic acid and the optical rotation can be retrieved from the experimental details, at least no misinformation is spread. We therefore suggest to use the prefix for the optical rotation only.

### Cytotoxic Effects

In our previous study, we investigated the effect of copaiba diterpenoids against A-375, HepG2, HT-29, HCT-116, A-549 and MDA-MB-231 cancer cell lines as well as non-cancerous HaCaT cell lines resulting in weak to moderate non-selective cytotoxic effects with IC_50_ values of 66.2 to 96.4 μg/ml for (−)-polyalthic acid (**1**) and 50.2 to 79.6 μg/ml for kaurenoic acid (**2**) (Pfeifer Barbosa et al. 2019). Thus, the effect of derivatising either the carboxyl group or the exocyclic methylene group was the main aim of the present study. In a first screening at a concentration of 100 μg/ml no effects for derivatives without the exocyclic methylene group (**1c** and **2c**–**2e**) were observed (Table S1). Compounds **2d** and **2e** were earlier tested on four different cancer cell lines (K562, HL-60, MDA-MB435 and SF295) as well as on healthy peripheral blood mononuclear cells and compared with kaurenoic acid (Cavalcanti et al. 2009). Also here, the cytotoxic effect disappeared after derivatisation; wherefore, the authors hypothesised that the exocyclic methylene group is crucial for the cytotoxic effect.

However, also derivatisation of the carboxyl group (compounds **1a**, **1b**, **2a** and **2b**) did not result in total growth inhibition at 100 μg/ml, but rather affected healthy HaCaT cells at this concentration. Because of the only low cytotoxic activity of compounds **1** and **2** found in our previous study and the not too promising results of the preliminary screening, we decided to focus on the antimicrobial effects of our compounds.

### Antibacterial Effects

Due to the strong effects of (−)-polyalthic acid (**1**) and especially kaurenoic acid (**2**) against *E. faecium* and methicillin-resistant *Staphylococcus aureus* (MRSA) (Pfeifer Barbosa et al. 2019), additional assays on *Klebsiella pneumoniae*, *Acinetobacter baumannii*, *Pseudomonas aeruginosa* and *Escherichia coli* as well as phytopathogens *Pseudomonas syringae*, *Xanthomonas campestris*, *Erwinia amylovora* and *Ralstonia solanacearum* were included in our investigations (Table S2). However, at a concentration of 100 μg/ml, activity of compounds **1** and **2** as well as their derivatives against the additionally investigated organisms was rather low or non-existent in contrast to the already known effects against *E. faecium* and MRSA. But also against the latter two strains derivatisations did not lead to an improved activity (Table 2). Here, only compound **2c** showed (at least moderate) effects, with IC_50_ values of 59.7 μg/ml against *E. faecium* and 34.7 μg/ml against MRSA. In contrast, (−)-polyalthic acid (**1**) and kaurenoic acid (**2**) showed IC_50_ values of 8.5 and 2.3 μg/ml against *E. faecium* as well as 8.9 and 3.4 μg/ml against MRSA, respectively. Thus, both the carboxyl group and the exocyclic methylene group seem to be pivotal for the activity against these two clinically relevant strains.

**Table 2:** Antibacterial effects of (−)-polyalthic acid (**1**), kaurenoic acid (**2**) and their derivatives. The IC_50_ values are in μg/ml. Positive controls were ampicillin (*Enterococcus faecium*) and chloramphenicol (MRSA)

	*E. faecium*	MRSA
Compound **1**	8.5 ± 0.4	8.9 ± 0.8
Compound **1a**	> 100	> 100
Compound **1b**	> 100	> 100
Compound **1c**	> 100	> 100
Compound **2**	2.3 ± 0.2	3.4 ± 0.2
Compound **2a**	> 100	> 100
Compound **2b**	> 100	> 100
Compound **2c**	59.7 ± 0.1	34.7 ± 2.5
Compound **2d**	> 100	> 100
Compound **2e**	> 100	> 100
Positive control	0.4 ± 0.1	1.2 ± 0.1

### Antifungal Effects

(−)-Polyalthic acid (**1**) was the most potent compound against *Trichopyhton* species dermatophytes in our previous study, with IC_50_ values of 6.8 μg/ml against *T. rubrum* and 4.3 μg/ml against *T. mentagrophytes*, respectively (Pfeifer Barbosa et al. 2019), while kaurenoic acid (**2**) only showed weak (70.8 μg/ml) to moderate effects (15.5 μg/ml) against these two strains (Table 3). Derivatisation of the two natural products this time revealed a different picture, leading to one equally potent (**1b**) and another still moderately active compound (**1a**) against *T. rubrum*, with IC_50_ values of 7.7 μg/ml for (−)-polyalthic acid methyl ester (**1b**) and 13.8 μg/ml for (−)-polyalthic acid *N*,*N*-diethylamide (**1a**), respectively. Notably, this effect was not observed against *T*. *mentagrophytes*, where the IC_50_ value was increased by the factor five. For kaurenoic acid (**2**), a similar picture was observed. Here, amidation (**2a**) and esterification (**2b**) yielded even more active compounds against *T. rubrum*, though on an overall higher level. Still, also against *T*. *mentagrophytes* the activity was drastically reduced by derivatisation of the carboxyl group. Of the remaining derivatives, only compound **1c** showed activity, with an IC_50_ of 61.8 μg/ml against *T. rubrum* and 31.9 μg/ml against *T*. *mentagrophytes*.

**Table 3:** Antifungal effects of (−)-polyalthic acid (**1**), kaurenoic acid (**2**) and their derivatives. The IC_50_ values are in μg/ml. Positive controls were clotrimazole (*Trichophyton rubrum* and *T. mentagrophytes*) and amphotericin B (*Cryptococcus neoformans*)

	*T. rubrum*	*T. mentagrophytes*	*C. neoformans*
Compound **1**	6.8 ± 0.1	4.3 ± 0.1	11.2 ± 0.3
Compound **1a**	13.8 ± 3.0	23.5 ± 0.6	9.5 ± 0.4
Compound **1b**	7.7 ± 0.6	21.8 ± 0.9	11.0 ± 4.9
Compound **1c**	61.8 ± 1.3	31.9 ± 1.6	> 100
Compound **2**	70.8 ± 1.6	15.5 ± 0.7	> 100
Compound **2a**	51.5 ± 7.0	> 100	> 100
Compound **2b**	53.0 ± 6.2	> 100	> 100
Compound **2c**	> 100	> 100	> 100
Compound **2d**	> 100	> 100	> 100
Compound **2e**	> 100	> 100	> 100
Positive control	0.1 ± 0.0	0.1 ± 0.0	0.6 ± 0.1

**Fig. 1:**
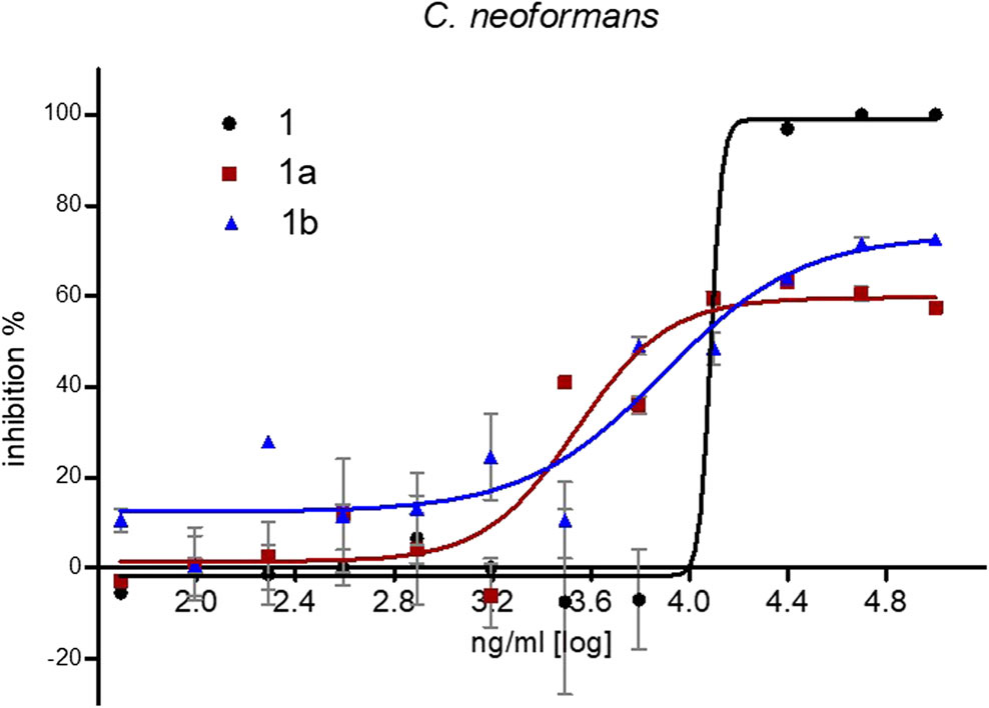
IC_50_ curves compounds **1**, **1a** and **1b** against *Cryptococcus neoformans*

The compounds were furthermore investigated for their inhibitory potential against two yeasts, *Candida albicans* and *Cryptococcus neoformans*. While the activity against *C. albicans* was negligible (Table S3), the results against *C. neoformans* revealed interesting findings for compounds **1**, **1a** and **1b**. All three compounds showed similar IC_50_ values, which were in the range of 9.5 to 11.2 μg/ml (Table 3). However, below this concentrations the activity of (−)-polyalthic acid (**1**) decreased rapidly in contrast to its two non-acidic derivatives (**1a** and **1b**) (Fig. 1).

## Conclusion

(−)-Polyalthic acid (**1**) and kaurenoic acid (**2**) as well as their semi-synthetic derivatives (**1a**–**1c** and **2a**–**2e**) were investigated for their cytotoxic, antibacterial and antifungal properties, with different outcomes. While the previously discussed importance of an exocyclic methylene group for the cytotoxic effect was also apparent in our study, we furthermore found that both the exocyclic methylene and the carboxylic acid group were necessary for the effect against *E. faecium* and MRSA.

However, different results were obtained with regard to the antifungal activity of the two natural products and their derivatives. For *T. mentagrophytes*, the picture was somehow similar to the antibacterial assays, where the best antifungal activity was achieved when both an exocylic methylene group and a carboxylic acid were present. In contrast, the presence of an carboxyl group was not required for activity against *C. neoformans* and *T. rubrum*, as displayed by the similar IC_50_ values of **1**, **1a** and **1b**. However, the low IC_50_ value of compound **1c** clearly shows that at least the methylene group is crucial for the effect.

Besides gaining further insights into differential biological activities and some SARs for copaiba diterpenoids, we present a novel semi-synthetic derivative of (−)-polyalthic acid. In the course of verifying the relative configuration of the new compound, we discovered ambiguities regarding the stereochemistry of polyalthic acid by two commonly used databases, which also led to incorrect chemical structures in several publications. We thus suggest to only use the terms (+)- and (−)-polyalthic acid to differentiate between the two enantiomers in order to hopefully avoid such mistakes in future publications.

## Electronic supplementary material

ESM 1 (PDF 648 kb)
