# Supplementary material for: Biological Activities of Two Major Copaiba Diterpenoids and Their Semi-synthetic Derivatives
Source: Rev Bras Farmacogn. 2020 Feb 21;30(1):18–27. doi: 10.1007/s43450-020-00002-y (PMC7222050; doi:10.1007/s43450-020-00002-y)
Supplement: Supplementary file 1 — (PDF 648 kb) [file 43450_2020_2_MOESM1_ESM.pdf]

Supporting information

Biological activities of two major copaiba diterpenoids and their semi-synthetic derivatives

Serhat Sezai Çiçek[a,*], Arlette Wenzel-Storjohann[b], Ulrich Girreser[c], Deniz Tasdemir[b,d]

[a] Pharmazeutisches Institut, Abteilung Pharmazeutische Biologie, Christian-Albrechts-Universität zu Kiel, Gutenbergstraße 76, 24118 Kiel, Germany

[b] GEOMAR Centre for Marine Biotechnology, Research Unit Marine Natural Products Chemistry, GEOMAR Helmholtz Centre for Ocean Research Kiel, Am Kiel-Kanal 44, 24106 Kiel, Germany

[c] Pharmazeutisches Institut, Abteilung Pharmazeutische und Medizinische Chemie, Christian-Albrechts-Universität zu Kiel, Gutenbergstraße 76, 24118 Kiel, Germany

[d] Christian-Albrechts-Universität zu Kiel, 24118 Kiel, Germany

* Corresponding author. E-mail address: scicek@pharmazie.uni-kiel.de.

Table of content

Figure S1: High-resolution MS spectrum of compound **1c**

Figure S2: ^{1}H NMR spectrum of compound **1c** measured in methanol-d_4

Figure S3: ^{13}C NMR spectrum of compound **1c** measured in methanol-d_4

Figure S4: H,H COSY spectrum of compound **1c** measured in methanol-d_4

Figure S5: HSQC spectrum of compound **1c** measured in methanol-d_4

Figure S6: HMBC spectrum of compound **1c** measured in methanol-d_4

Figure S7: ^{1}H NMR spectrum of compound **1c** measured in DMSO-d_6

Figure S8: ^{13}C NMR spectrum of compound **1c** measured in DMSO-d_6

Figure S9: H,H COSY spectrum of compound **1c** measured in DMSO-d_6

Figure S10: HSQC spectrum of compound **1c** measured in DMSO-d_6

Figure S11: HMBC spectrum of compound **1c** measured in DMSO-d_6

Figure S12a: NOESY spectrum of compound **1c** measured in DMSO-d_6

Figure S12b: NOESY spectrum of compound **1c** in the range of 0.5 to 3.6 ppm measured in DMSO-d_6

Table S1: Cytotoxic effects at a concentration of 100 µg/mL given in % of inhibition

Table S2: Antibacterial effects at a concentration of 100 µg/mL given in % of inhibition

Table S3: Antifungal effects at a concentration of 100 µg/mL and corresponding IC_{50} values

Fig. S1:

High-resolution MS spectrum of compound **1c**

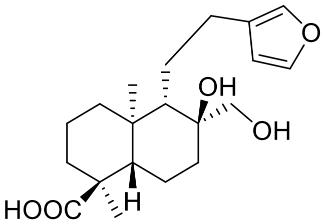

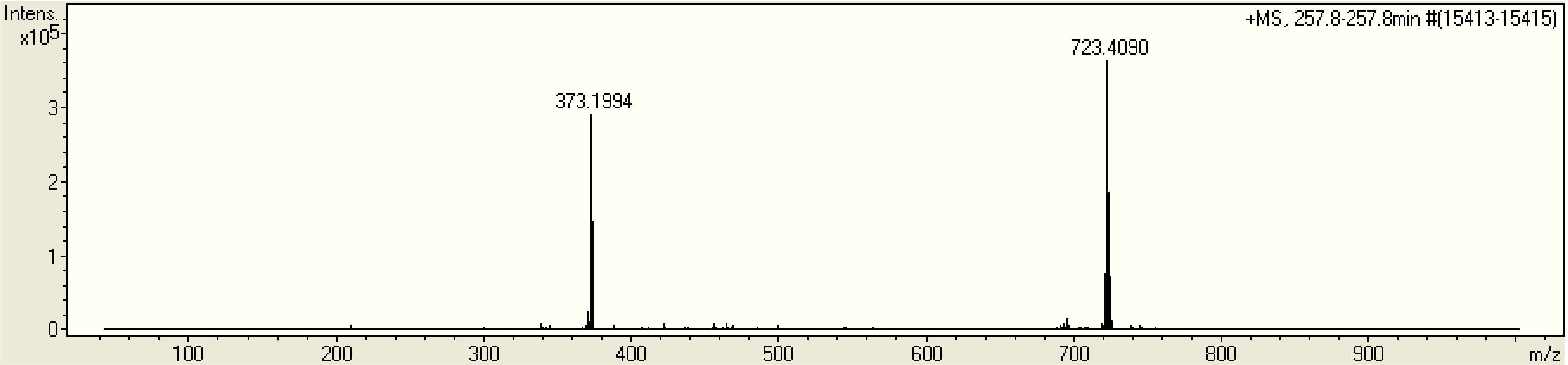

Fig. S2:

^{1}H NMR spectrum of compound **1c** measured in methanol-d_4

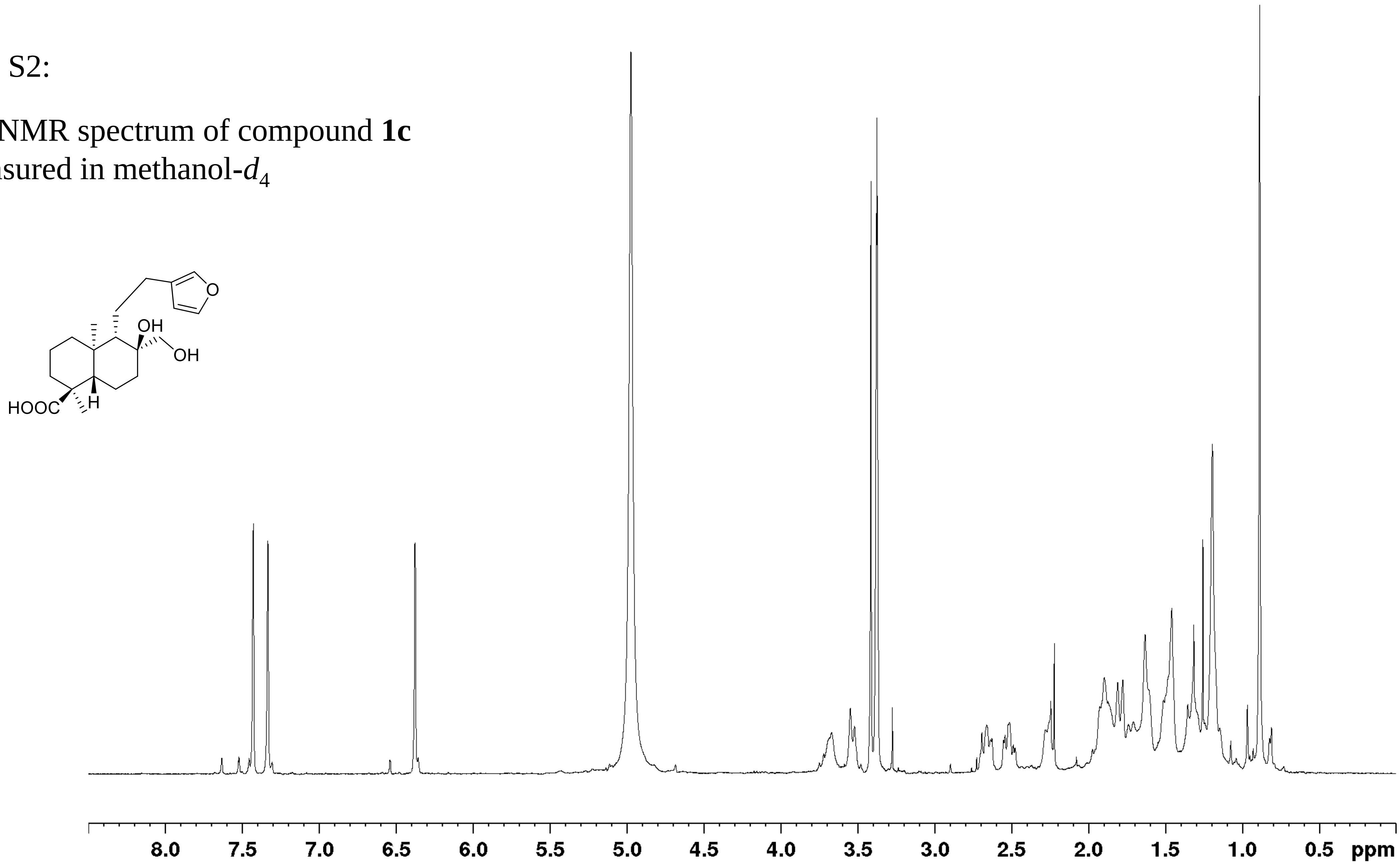

Fig. S3:

^{13}C NMR spectrum of compound **1c** measured in methanol-d_4

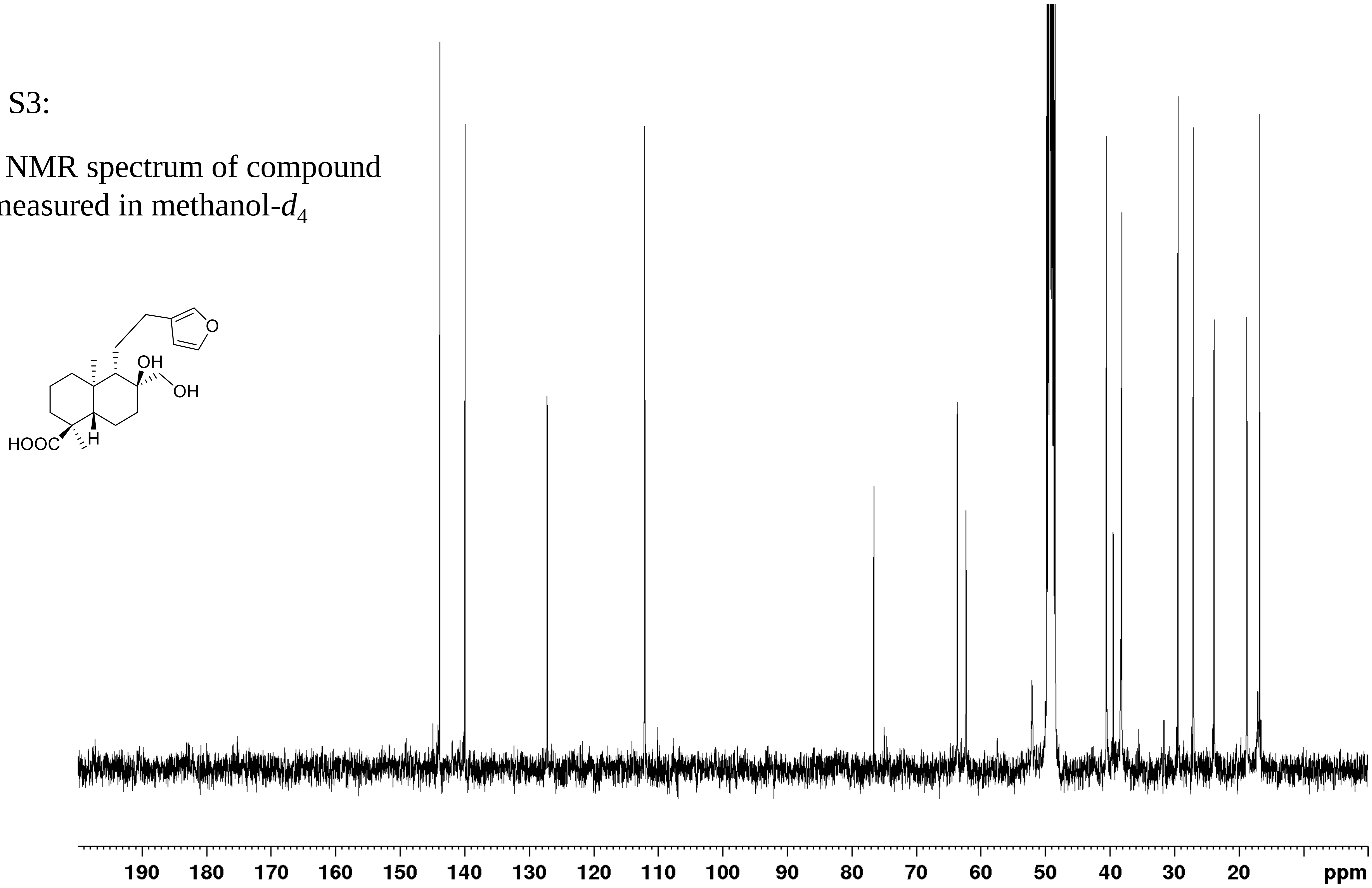

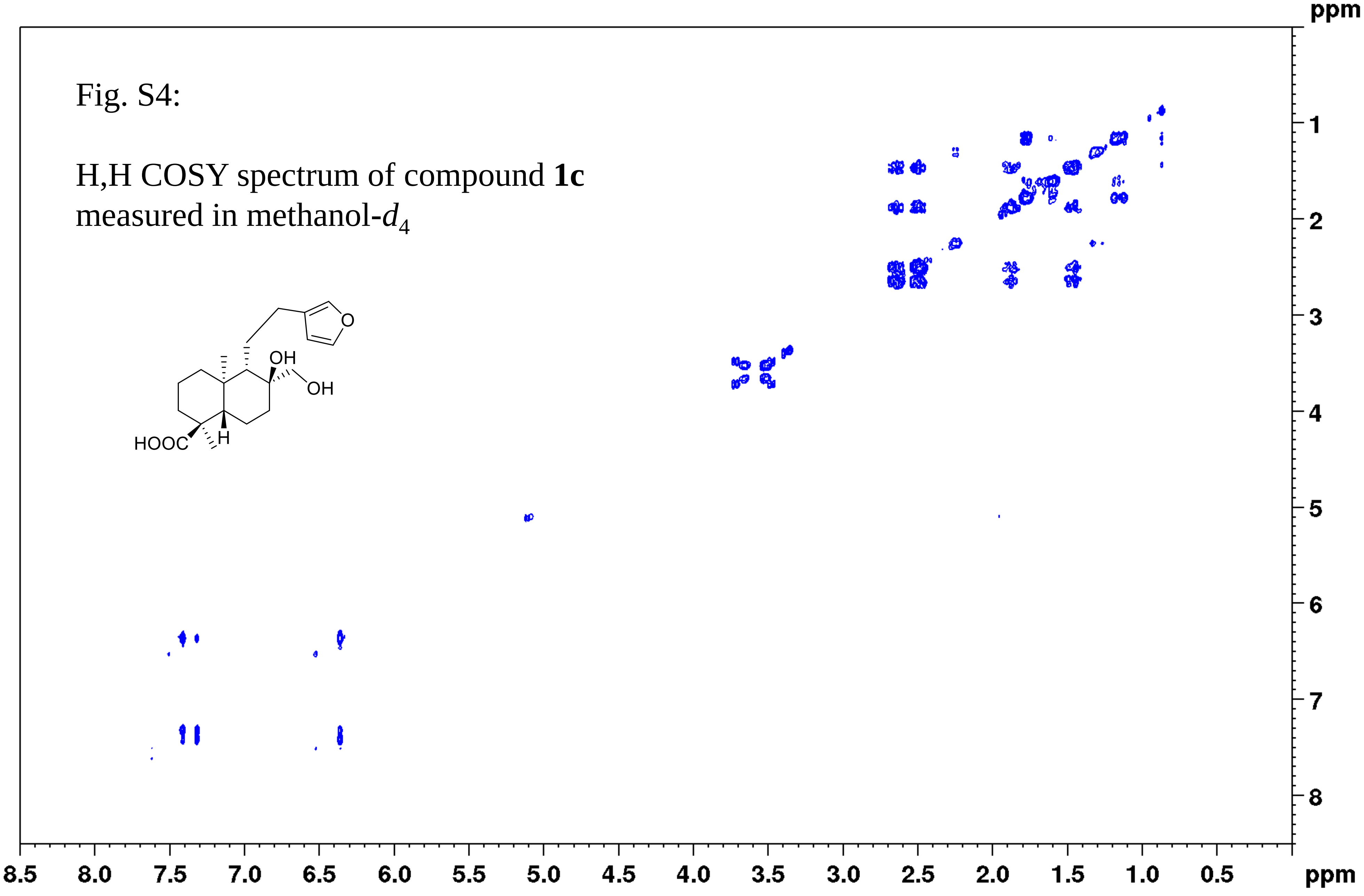

Fig. S4:

H,H COSY spectrum of compound **1c** measured in methanol-d_4

Fig. S5:

HSQC spectrum of compound **1c** measured in methanol-d_4

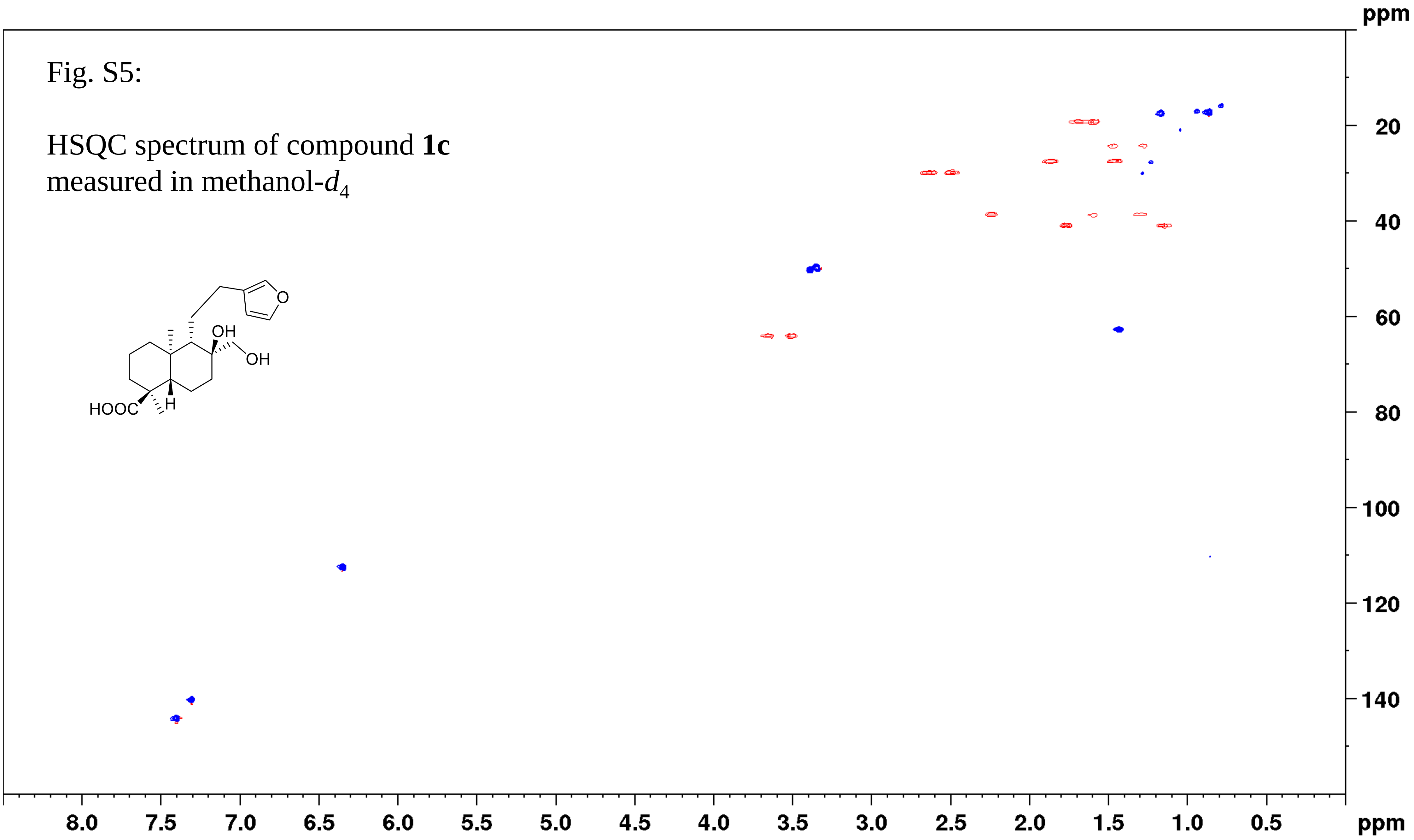

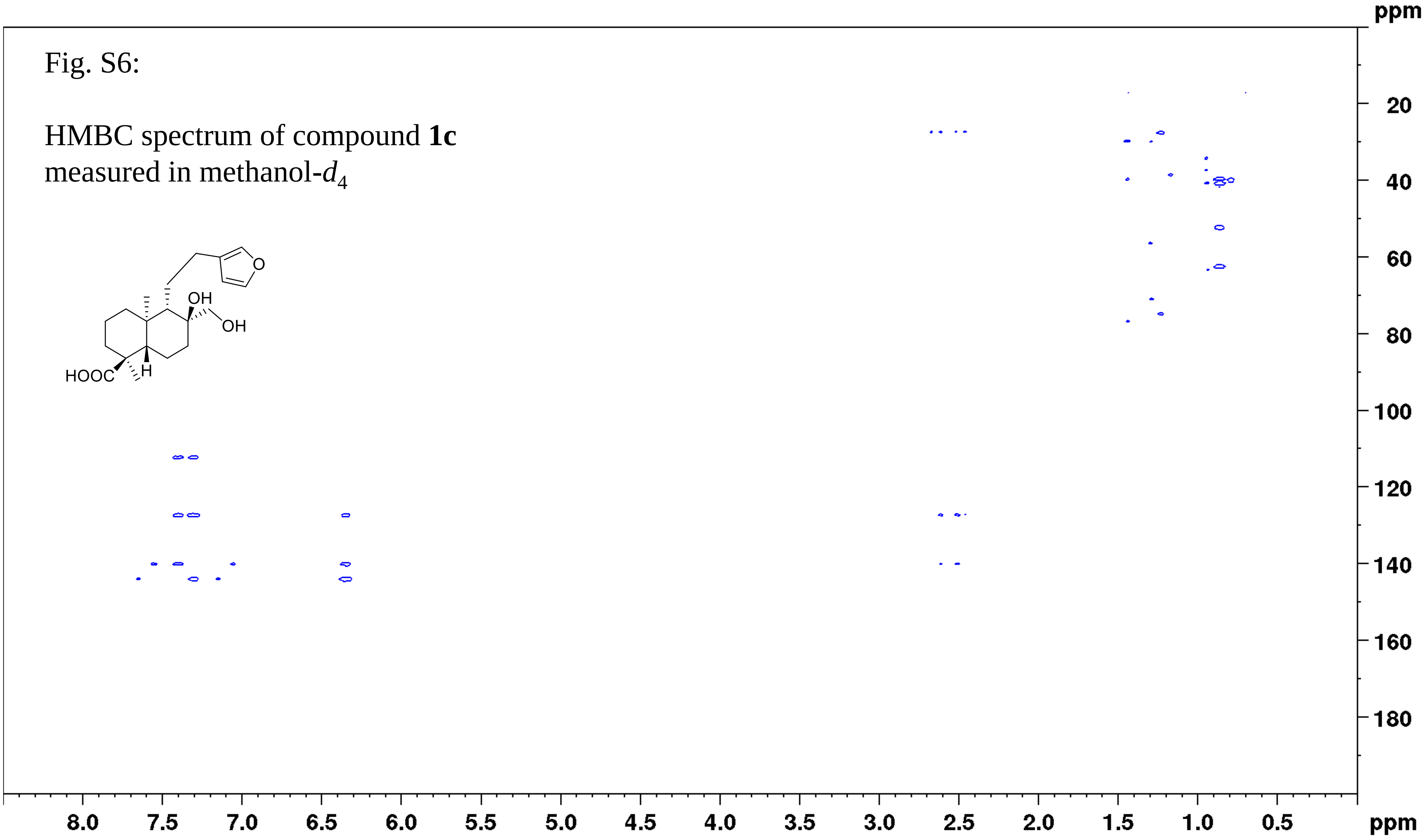

Fig. S6:

HMBC spectrum of compound **1c** measured in methanol-d_4

Fig. S7:

^{1}H NMR spectrum of compound **1c** measured in DMSO-d_6

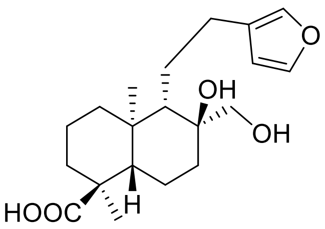

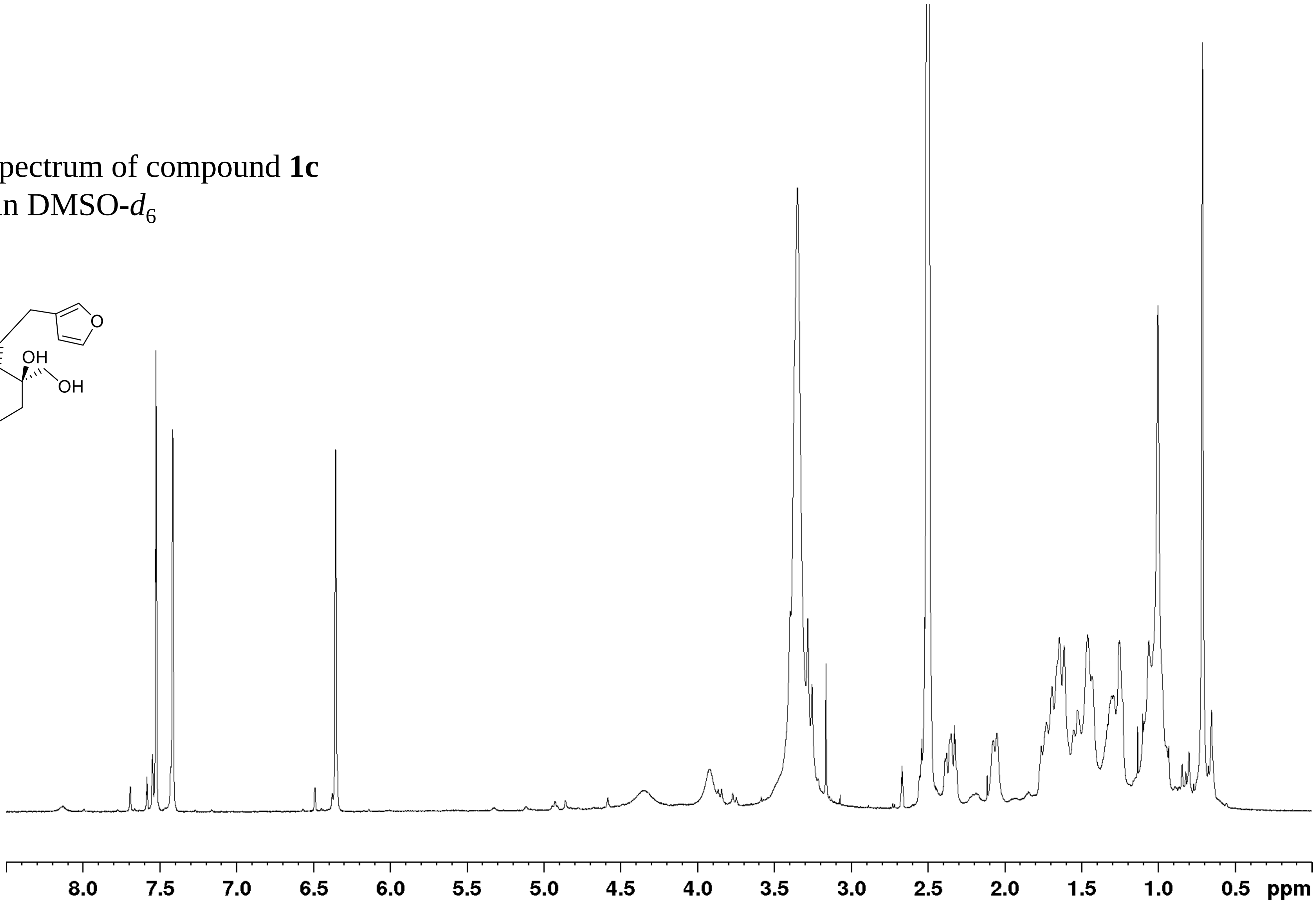

Fig. S8:

^{13}C NMR spectrum of compound **1c** measured in DMSO-d_6

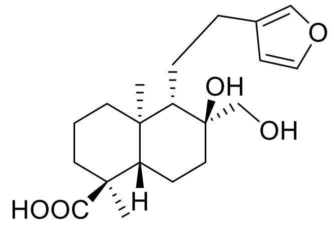

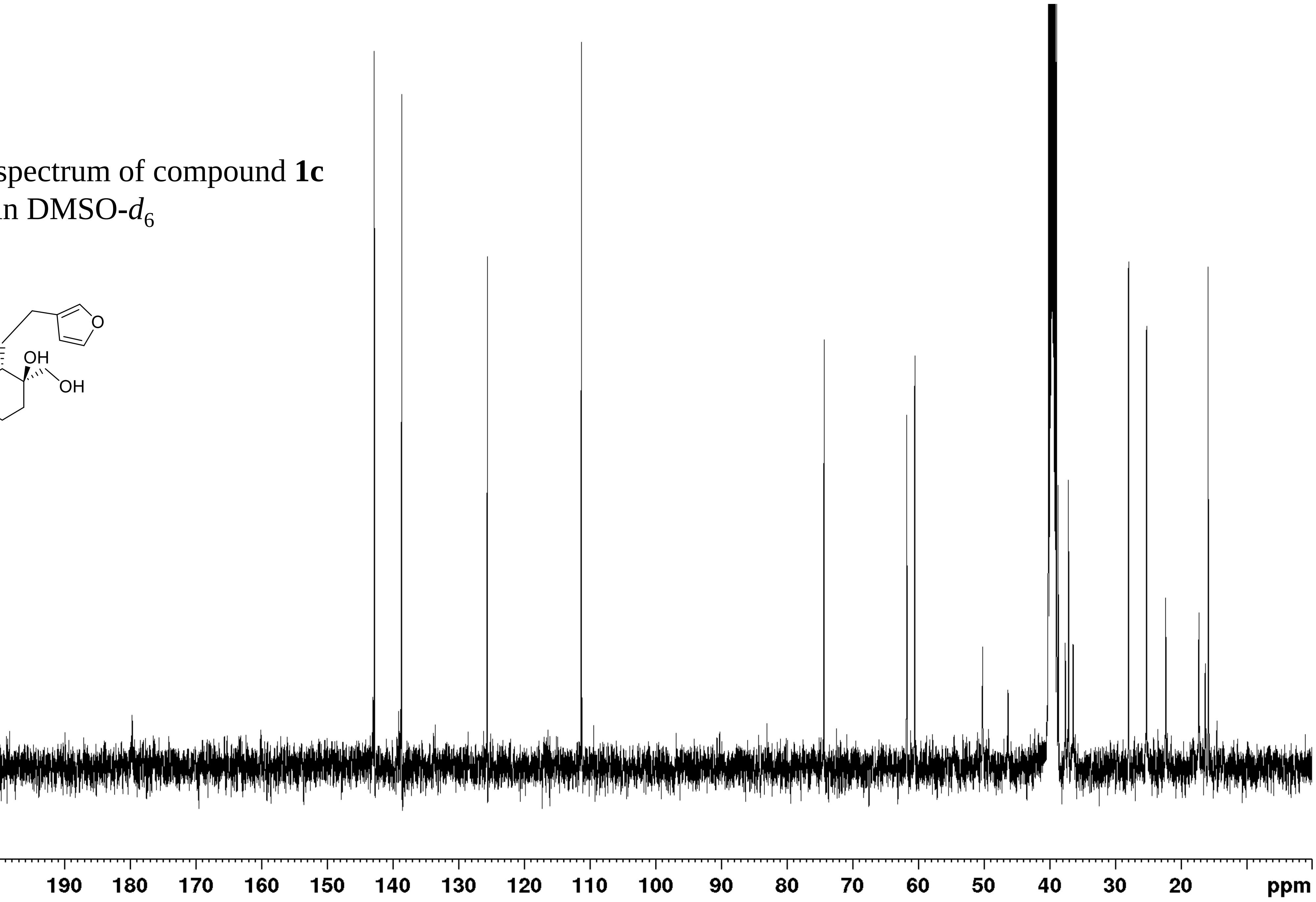

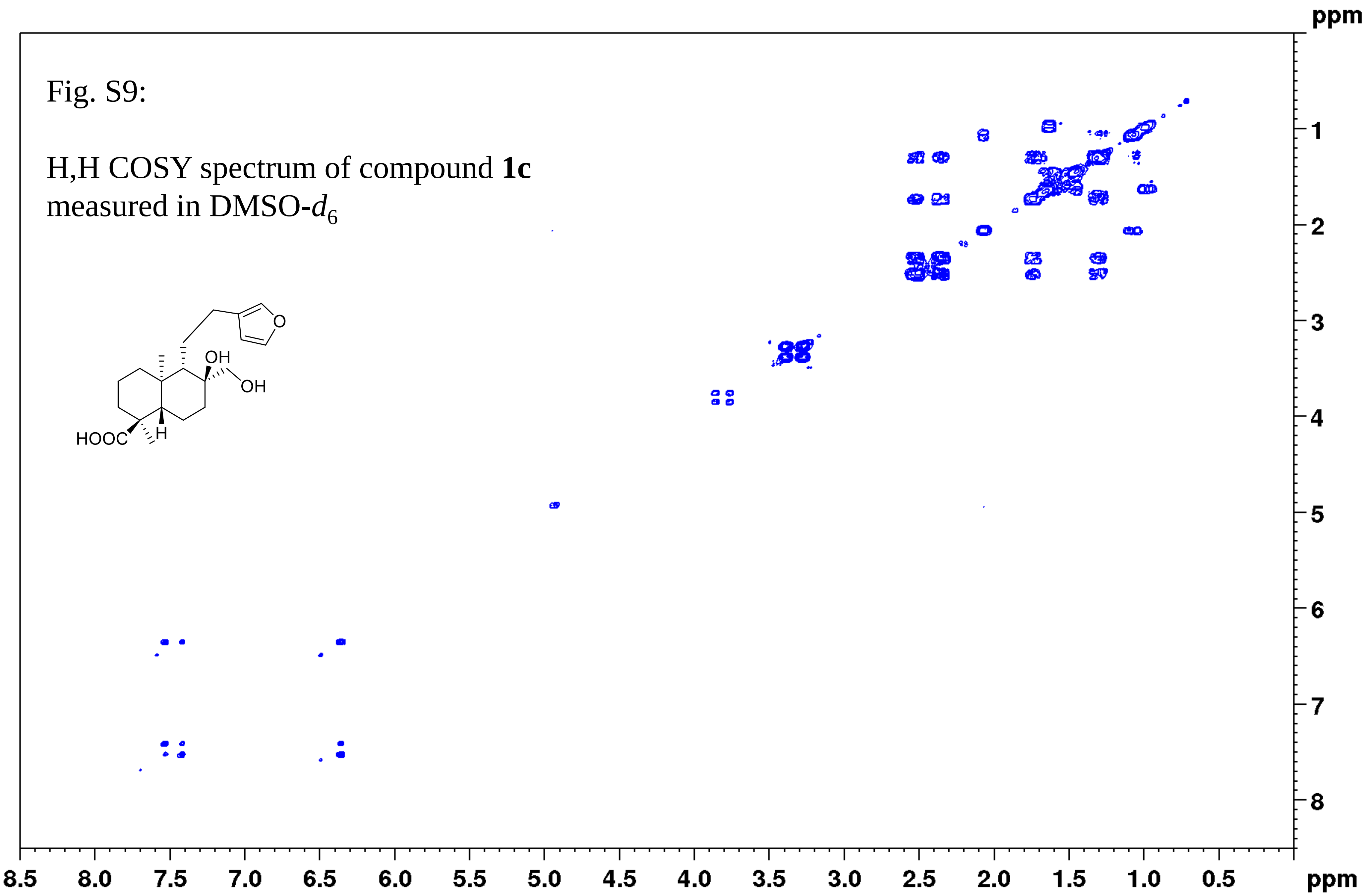

Fig. S9:

H,H COSY spectrum of compound **1c** measured in DMSO-d_6

Fig. S10:

HSQC spectrum of compound **1c** measured in DMSO-d_6

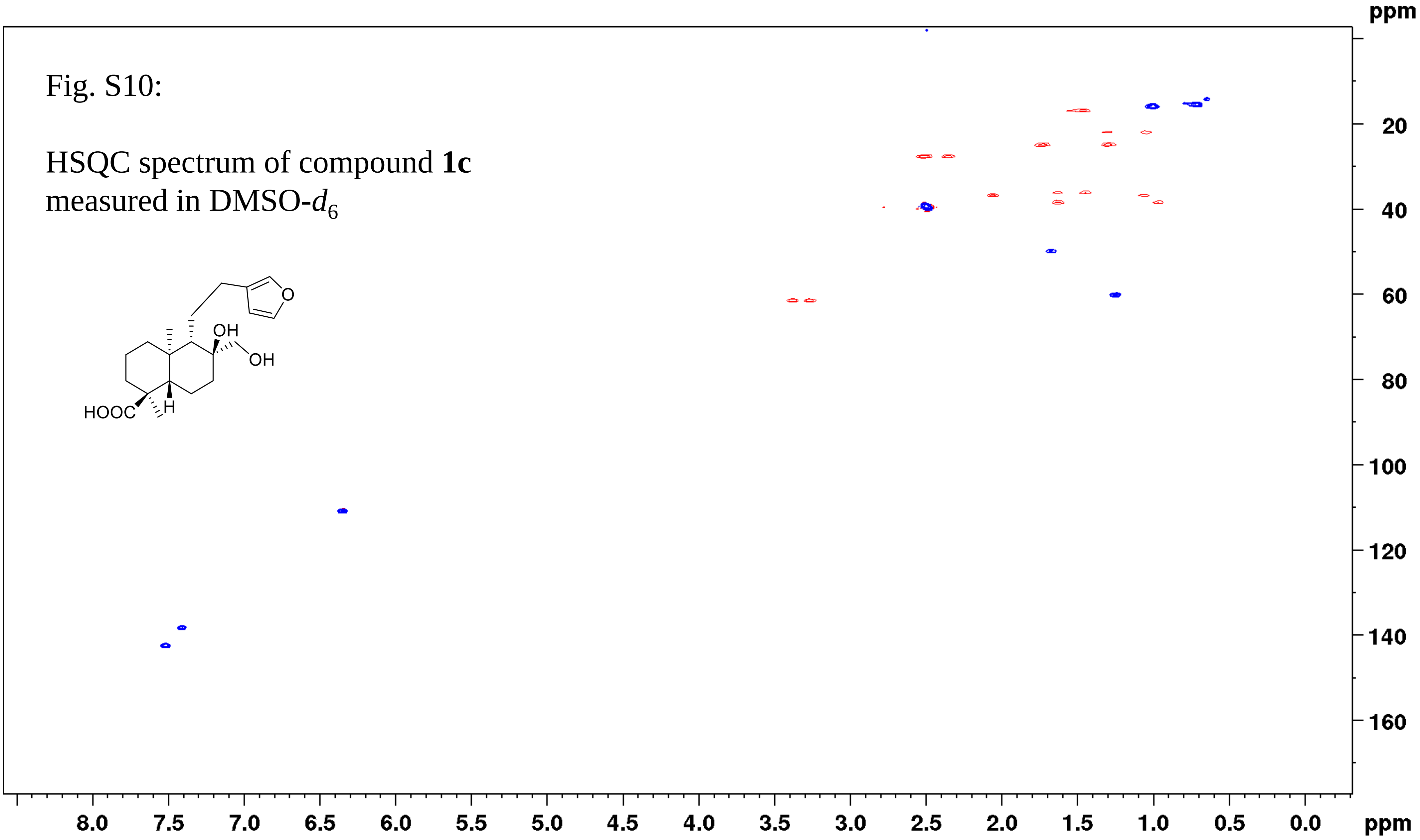

Fig. S11:

HMBC spectrum of compound **1c** measured in DMSO-d_6

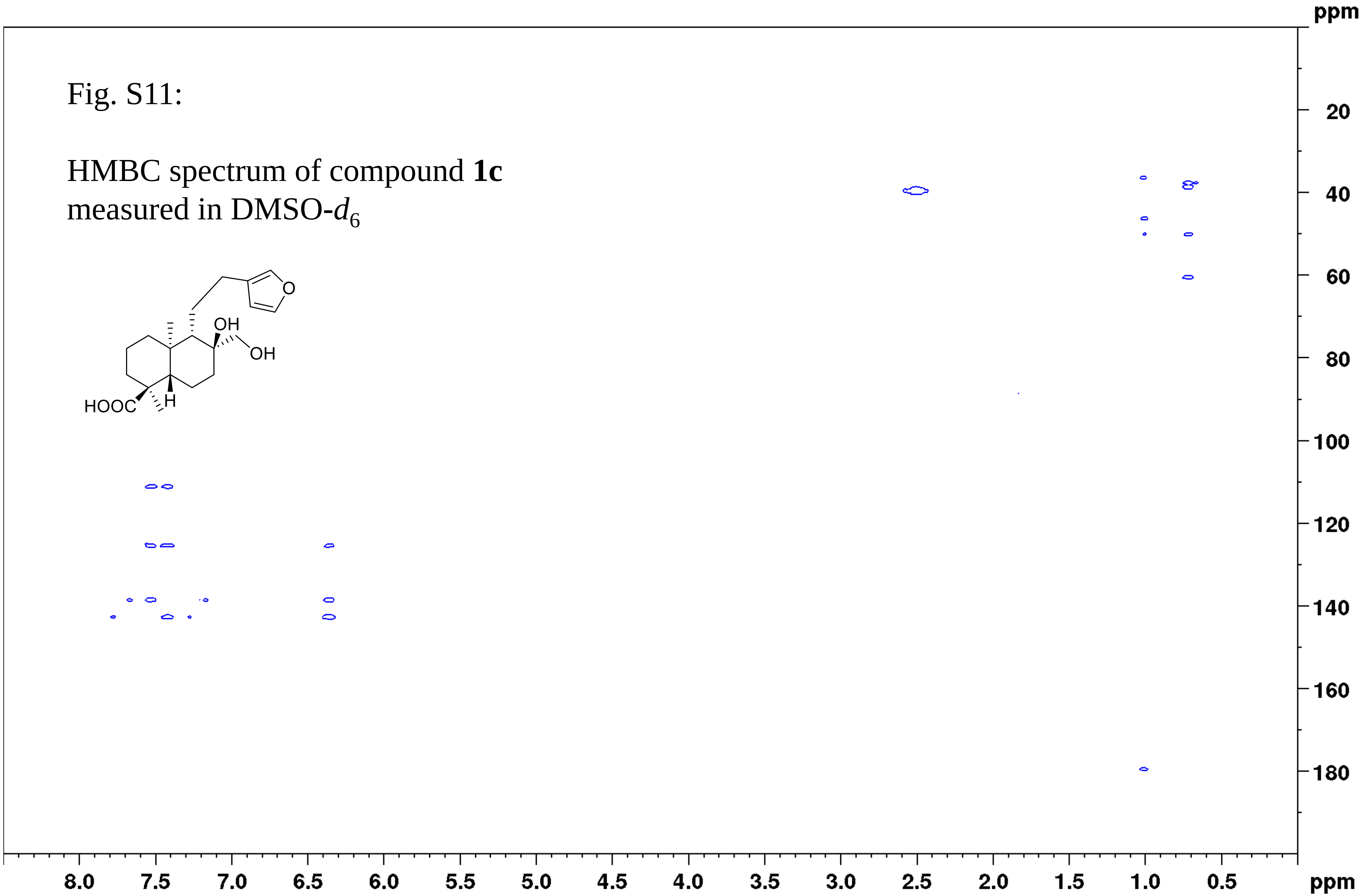

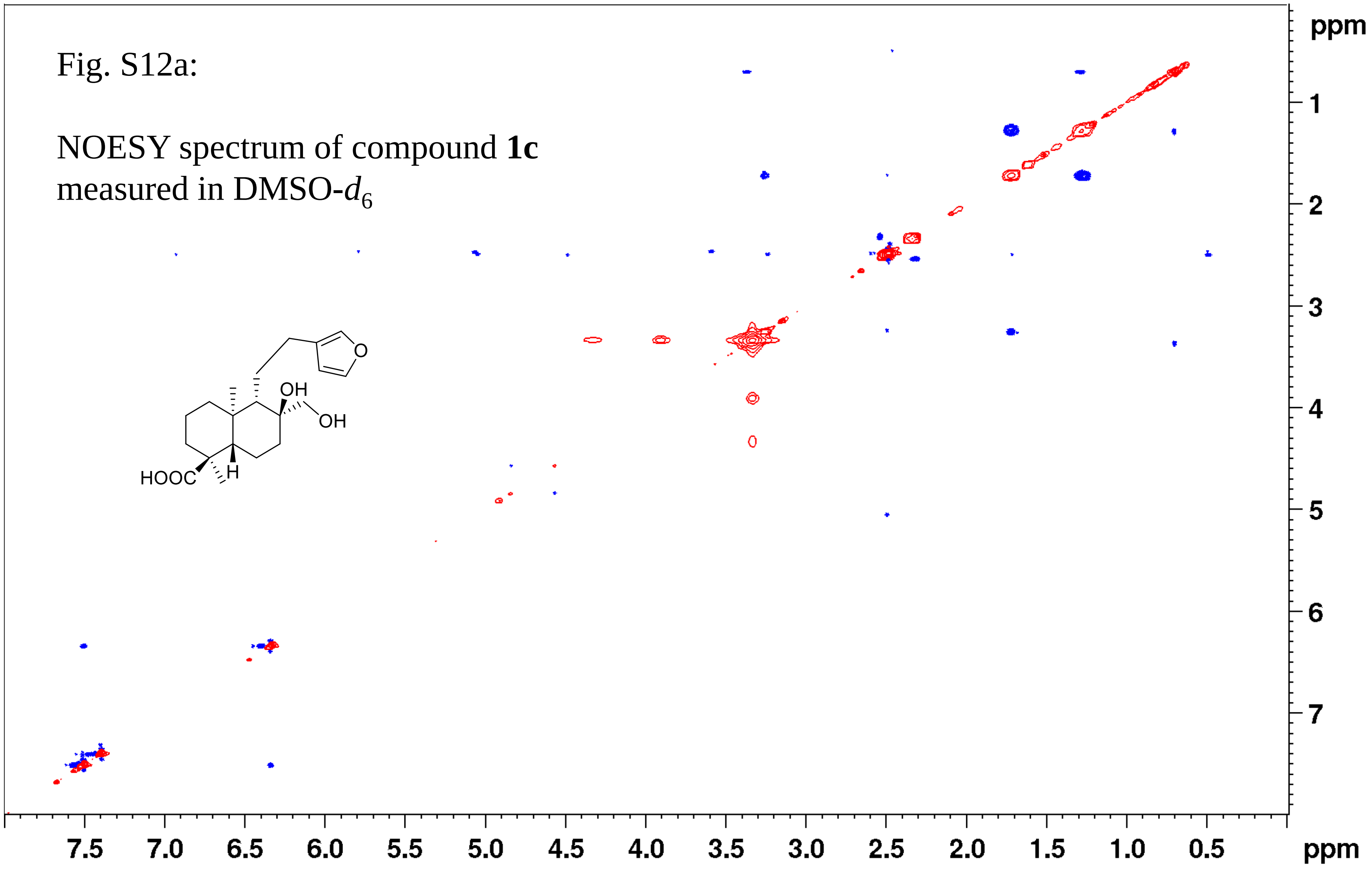

Fig. S12a:

NOESY spectrum of compound **1c** measured in DMSO-d_6

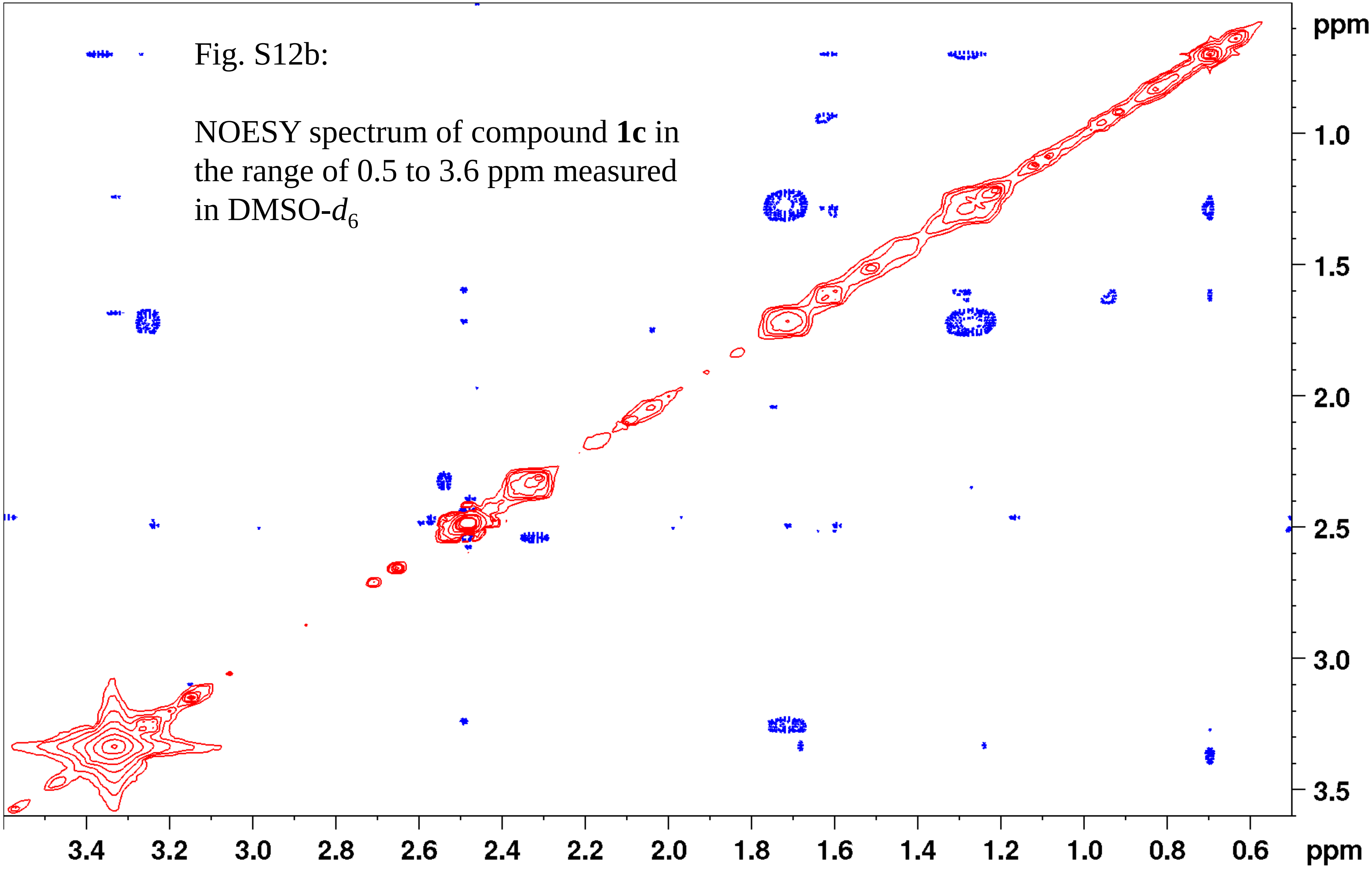
Fig. S12b:
NOESY spectrum of compound **1c** in the range of 0.5 to 3.6 ppm measured in DMSO-d_6
ppm
1.0
1.5
2.0
2.5
3.0
3.5
3.4
3.2
3.0
2.8
2.6
2.4
2.2
2.0
1.8
1.6
1.4
1.2
1.0
0.8
0.6
ppm

Table S1

Cytotoxic effects at a concentration of 100 μg/mL given in % of inhibition. Doxorubicin was used as positive control. Values below 20% inhibition are expressed as "–".

	A-375	Hep-G2	HT-29	HCT-116	A-549	MDA-MB-231	HaCaT
Compound **1**	83	64	–	32	83	–	–
Compound **1a**	90	86	92	80	90	81	98
Compound **1b**	73	39	70	51	73	44	97
Compound **1c**	–	–	–	–	–	–	–
Compound **2**	97	98	87	93	97	31	96
Compound **2a**	51	–	–	–	51	–	67
Compound **2b**	46	–	–	25	46	70	89
Compound **2c**	–	–	–	–	–	–	–
Compound **2d**	–	–	–	–	–	–	–
Compound **2e**	–	–	–	–	–	–	–
Positive control	80	77	82	70	80	72	67

Table S2

Antibacterial effects at a concentration of 100 µg/mL given in % of inhibition. Positive controls were chloramphenicol (*K. pneumonia, A. baumannii, E. coli, P. syringae, X. campestris* and *E. amylovora*), polymyxin B (*P. aeruginosa*) and tetracycline (*R. solanacearum*). Values below 20% inhibition are expressed as "–".

	K. pneumoniae	*A. baumannii*	*P. aeruginosa*	*E. coli*	*P. syringae*	*X. campestris*	*E. amylovora*	*R. solanacearum*
Compound **1**	–	54	–	–	–	71	–	38
Compound **1a**	–	–	44	–	–	–	–	53
Compound **1b**	–	–	37	–	–	–	–	62
Compound **1c**	–	–	–	–	–	–	–	–
Compound **2**	–	–	–	–	–	67	–	35
Compound **2a**	–	–	32	–	–	–	–	47
Compound **2b**	–	–	–	–	–	–	–	25
Compound **2c**	–	–	–	–	–	23	–	63
Compound **2d**	–	–	–	–	–	–	–	39
Compound **2e**	–	–	–	–	25	21	–	46
Positive control	97	100	100	77	95	96	84	97

Table S3

Antifungal effects at a concentration of 100 μg/mL and corresponding IC_{50} values (in μg/mL). Positive controls were clotrimazole (*T. rubrum* and *T. mentagrophytes*), nystatin (*C. albicans*) and amphotericin B (*C. neoformans*). Values below 20% inhibition are expressed as "–".

	T. rubrum	*T. mentagrophytes*	*C. albicans*	*C. neoformans*
Compound **1**	100	100	32	100
Compound **1a**	61	84	39	58
Compound **1b**	82	100	35	73
Compound **1c**	87	97	–	–
Compound **2**	62	89	–	37
Compound **2a**	62	31	39	48
Compound **2b**	63	36	–	41
Compound **2c**	–	49	–	32
Compound **2d**	–	30	–	32
Compound **2e**	–	42	–	34
Positive control	96	98	99	92
